# Intermittent Hypoxia Alters the Circadian Expression of Clock Genes in Mouse Brain and Liver

**DOI:** 10.3390/genes12101627

**Published:** 2021-10-16

**Authors:** Bala S. C. Koritala, Yin Yeng Lee, Shweta S. Bhadri, Laetitia S. Gaspar, Corinne Stanforth, Gang Wu, Marc D. Ruben, Lauren J. Francey, David F. Smith

**Affiliations:** 1 Division of Pediatric Otolaryngology-Head and Neck Surgery, Cincinnati Children’s Hospital Medical Center, Cincinnati, OH 45229, USA; bala.koritala@icloud.com (B.S.C.K.); shweta.bhadri@cchmc.org (S.S.B.); laetitia.gaspar@cnc.uc.pt (L.S.G.); stanforthc1@udayton.edu (C.S.); 2 Department of Pediatrics, Division of Human Genetics, Cincinnati Children’s Hospital Medical Center, Cincinnati, OH 45229, USA; yinyeng.lee@cchmc.org (Y.Y.L.); gang.wu@cchmc.org (G.W.); marc.ruben@cchmc.org (M.D.R.); lauren.francey@cchmc.org (L.J.F.); 3 Department of Pharmacology and Systems Physiology, University of Cincinnati College of Medicine, Cincinnati, OH 45267, USA; 4 Center for Neuroscience and Cell Biology, University of Coimbra, 3004-504 Coimbra, Portugal; 5 Department of Biology, College of Arts and Sciences, University of Dayton, Dayton, OH 45469, USA; 6 Department of Otolaryngology-Head and Neck Surgery, University of Cincinnati College of Medicine, Cincinnati, OH 45267, USA; 7 Division of Pulmonary Medicine, Cincinnati Children’s Hospital Medical Center, Cincinnati, OH 45229, USA; 8 The Sleep Center, Cincinnati Children’s Hospital Medical Center, Cincinnati, OH 45229, USA; 9 The Center for Circadian Medicine, Cincinnati Children’s Hospital Medical Center, Cincinnati, OH 45229, USA

**Keywords:** intermittent hypoxia, circadian rhythms, canonical clock genes

## Abstract

At least one-third of adults in the United States experience intermittent hypoxia (IH) due to health or living conditions. The majority of these adults suffer with sleep breathing conditions and associated circadian rhythm disorders. The impact of IH on the circadian clock is not well characterized. In the current study, we used an IH mouse model to understand the impact of IH on the circadian gene expression of the canonical clock genes in the central (the brain) and peripheral (the liver) tissues. Gene expression was measured using a Quantitative Reverse Transcription Polymerase Chain Reaction (qRT-PCR). CircaCompare was used to evaluate the differential rhythmicity between normoxia and IH. Our observations suggested that the circadian clock in the liver was less sensitive to IH compared to the circadian clock in the brain.

## 1. Introduction

Oxygen is an essential element required for the fundamental cellular processes. Insufficient (hypoxia) or excessive (hyperoxia) oxygen alters the functions of molecules, cells, tissues/organs, and can lead to death [1]. Intermittent hypoxia (IH) is a hallmark of multiple respiratory conditions including chronic obstructive pulmonary disease (COPD) and obstructive sleep apnea (OSA) [2,3]. A chronic exposure to IH may be associated with cognitive abnormalities, cardiovascular diseases, as well as metabolic and inflammatory disorders [4]. These comorbidities are also associated with disrupted sleep and misaligned circadian rhythms.

The respiratory patterns of oxygen are controlled by the circadian clock [5]. In turn, oxygen rhythmicity can synchronize the clock through hypoxia-inducible factors (HIFs) [6]. The key transcriptional activators of the hypoxia signaling pathway, HIFs, and the circadian clock components, BMAL1 and CLOCK, belong to the same family of transcription factors [7]. Therefore, HIFs and canonical clock genes can regulate one another and influence circadian rhythms [8]. Despite rodent studies demonstrating that IH impacts gene expression in a time-dependent and tissue-specific manner [9,10], little is known about the impact of IH on the circadian clock. In the current study, we modeled IH in mice to represent OSA and better understand the impact of IH on the circadian rhythms of the canonical clock genes. After the exposure to seven days of normoxia or IH, brain (central) and liver (peripheral) tissues were collected for the measurement of the circadian expression of 10 canonical clock genes including *Arntl*, *Clock*, *Cry1*, *Cry2*, *Per1*, *Per2*, *Per3*, *Npas2*, *Dbp*, and *Nr1d1*. Our results suggested that the clock in the liver was less sensitive to IH, whereas the clock in the brain had a higher sensitivity to IH exposure.

## 2. Materials and Methods

### 2.1. Study Design and Sample Collection

**Figure 1:**
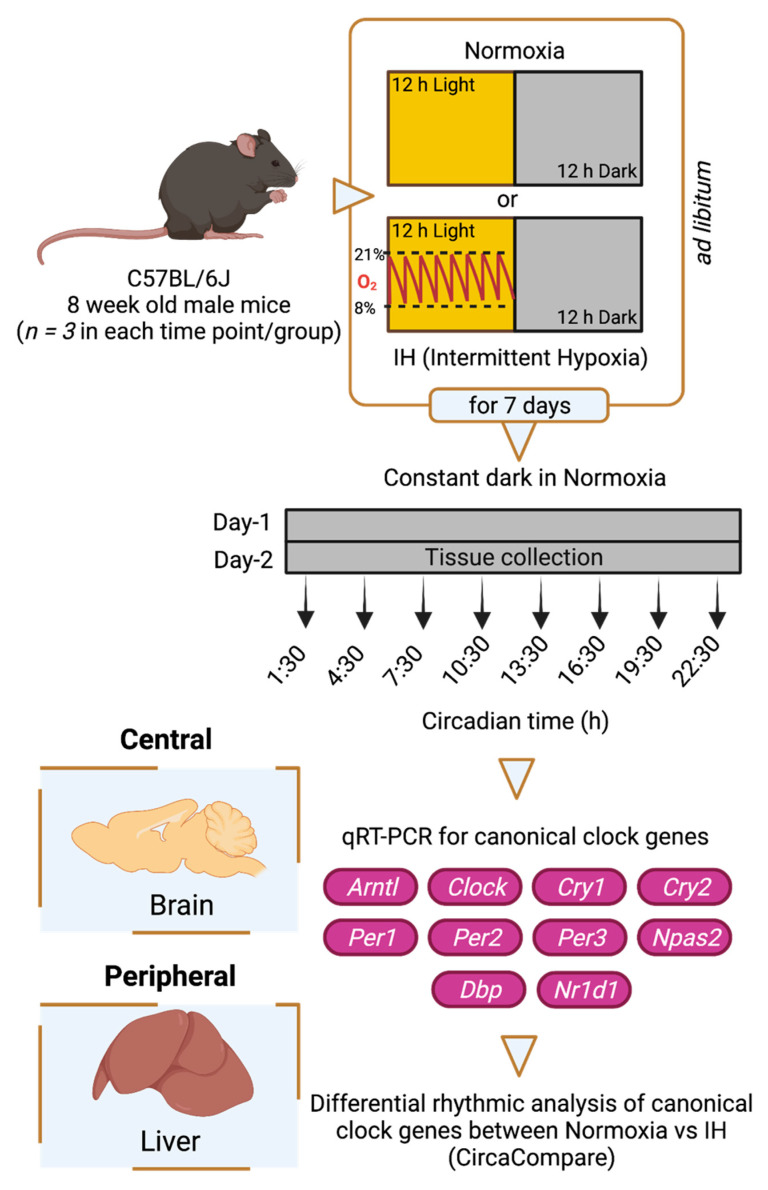
Study design. Six-week-old C57BL/6J male mice were entrained under LD 12:12 h for fourteen days. Animals were then exposed to a seven-day normoxia or intermittent hypoxia (IH) condition. Animals housed under IH received hypoxic events only in the 12 h light phase. After the seventh day of IH exposure, animals from both normoxic and IH conditions were housed under constant exposure to normoxia for two days in constant darkness. On the second day, central (brain) and peripheral (liver) tissues were collected over 24 h at three-hour intervals. Three animals (*n* = 3) were sacrificed at each time point in both conditions. All animals were subjected to ad libitum feeding and stringent temperature control. RNA was purified from these tissues, and the expression of canonical clock genes (*Arntl*, *Clock*, *Cry1*, *Cry2*, *Per1*, *Per2*, *Per3*, *Npas2*, *Dbp*, *Nr1d1*) was measured using Quantitative Reverse Transcription Polymerase Chain Reaction (qRT-PCR). The data were analyzed through CircaCompare to evaluate differential rhythmicity between conditions. This figure was generated through BioRender.com (accessed on 15 October 2021).

To understand the impact of IH on tissue-specific circadian rhythms, we used an IH model for C57BL/6J male mice that involved exposure to oxygen desaturation episodes ranging between 21% and 8% (BioSpherix OxyCycler A84XOV, Parish, NY, USA). Prior to a seven-day exposure to either normoxia or IH, six-week-old male mice (C57BL/6J) purchased from the Jackson Laboratory (#000664) were entrained under 12 h light and 12 h dark cycles (LD 12:12 h) for fourteen days under standard facility lighting. IH exposure (~50 episodes/h) occurred only in the inactive (light) phase of LD 12:12 h. After exposure, animals from both conditions were housed in constant darkness for two days under normoxic conditions. On the second day of constant darkness, brain and liver tissues were collected under red light every three hours for twenty-four hours (Figure 1). This protocol is a modification of our established model of IH [10]. Three animals (*n* = 3) were sacrificed at each time point in both normoxic and IH conditions. Tissues were snap frozen using liquid nitrogen and preserved at −80 °C for future gene expression analysis. All animals were subjected to ad libitum feeding and stringent temperature control.

### 2.2. Gene Expression Analysis

To extract RNA, frozen brain or liver tissues were homogenized in liquid nitrogen using mortar and pestle. Total RNA was purified using a Trizol-based RNA extraction method. A 1 mL volume of ice-cold TRIzol^TM^ reagent (Invitrogen^TM^, #15596018, Caslsbad, CA, USA) was added to ~100 mg of ground tissue. After mixing, 0.2 mL of chloroform (Fisher Chemical^TM^, #C298-500, Fair Lawn, NJ, USA) was added to the TRIzol tissue cocktail, mixed well, and incubated at room temperature for 3 min. Following centrifugation at 12,000× *g* for 15 min, the aqueous phase (top layer) was collected to a new tube, and an equal amount of isopropanol (Sigma Aldrich, #437522-4L, Saint Louis, MO, USA) was added. After a 10 min incubation at room temperature, samples were centrifuged at 12,000× *g* for 10 min, the supernatant was discarded, and the RNA pellet was washed three times with 70% ice-cold ethanol from 200 proof pure ethanol (Koptec, #V1016, King of Prussia, PA, USA). The RNA pellet was then air dried for 5–10 min and dissolved in 25 µL of UltraPure^TM^ DNase/RNase-Free Distilled Water (Invitrogen^TM^, #10977015, Carlsbad, CA, USA). The quantity and quality of RNA was measured based on the ratio of light absorbance at 260 and 230 nm (NanoDrop^®^ 1000 Spectrophotometer, Wilmington, DE, USA). After DNase-I (Invitrogen^TM^, #18068015, Carlsbad, CA, USA) treatment, cDNA was synthesized from RNA using Applied Biosystems^TM^ TaqMan^TM^ Reverse Transcription Reagents (Applied Biosystems^TM^, #N8080234, Carlsbad, CA, USA). Real-time (RT) PCR was performed using TaqMan^TM^ Fast Advanced Master Mix (Applied Biosystems^TM^, #N4444556, Carlsbad, CA, USA) in a 384-well QuantStudio^TM^ 5 Real-Time PCR System (Applied Biosystems^TM^, #A28140, Carlsbad, CA, USA). The qRT PCR reaction included denaturation (3 min at 95 °C) and 40 PCR cycles (15 s at 95 °C and 1 min at 60 °C). The results for each gene were normalized to the geometric mean expression of housekeeping genes, *Gapdh* and *Ppib*, by the ΔCt method and then scaled to the sample with the highest expression level (ΔΔCt) for time courses. The expression of the canonical clock genes (*Arntl*, *Clock*, *Cry1*, *Cry2*, *Per1*, *Per2*, *Per3*, *Npas2*, *Dbp*, and *Nr1d1*) in brain and liver were measured using TaqMan^®^ Gene Expression Assays (Applied Biosystems^TM^, #4331182, Carlsbad, CA, USA) (Table 1).

### 2.3. Analysis of Circadian Rhythms

CircaCompare [11], a non-linear curve fit method, was used to measure differential rhythmicity of canonical clock genes between normoxia and IH. Mean expression levels and standard deviations were calculated for each group separately. Measurements with 3 standard deviations from the mean were removed. We estimated loss or gain of rhythms and circadian properties including amplitude, phase, and mesor for each gene in two different conditions. Statistical significance was determined by *p* < 0.05.

## 3. Results

### 3.1. IH Alters Tissue-Specific Circadian Rhythms of Canonical Clock Genes

**Figure 2:**
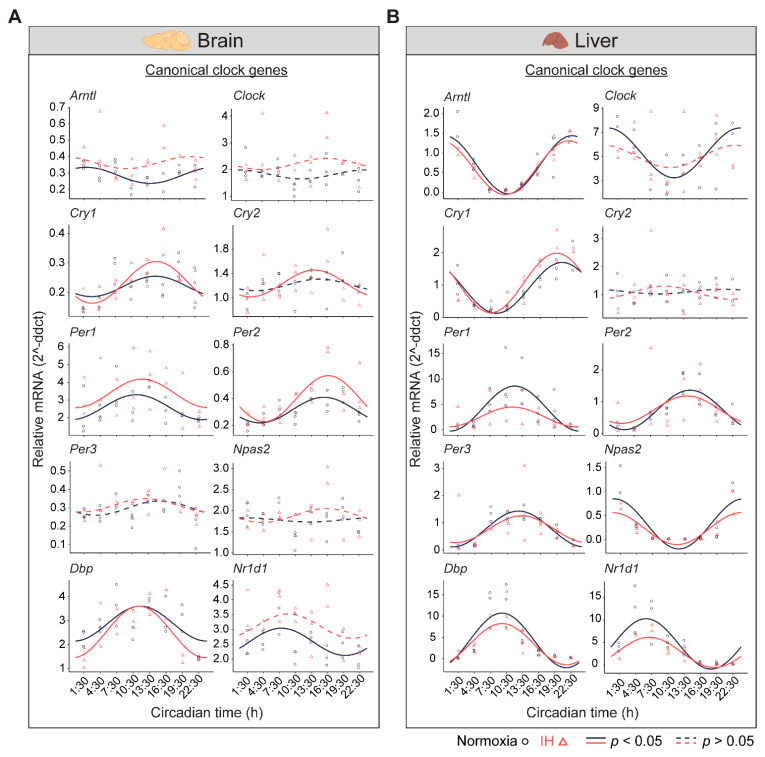
Circadian rhythms of canonical clock genes. (**A**) Brain and (**B**) Liver panels represent the expressions of canonical clock genes over 24 h on the second day of constant darkness after exposure to a normoxia (circles; black) and IH (triangles; red). Solid and dashed lines represent rhythmic (*p* < 0.05) and non-rhythmic (*p* > 0.05) expression patterns, respectively.

The central circadian clock in the brain regulates several physiological functions including breathing, hunger, sleep, and body temperature. Compared to other organs, the brain is more sensitive to changes in oxygen levels [12,13]. Therefore, it is important to understand the influence of IH on the clocks in the brain. We measured the differential rhythms of the canonical clock genes between normoxia versus IH (Figure 2A). We observed rhythms in *Cry1*, *Per1*, *Per2*, and *Dbp* in both conditions. In contrast, the *Arntl*, *Cry2*, and *Nr1d1* rhythms were altered by the exposure to IH. *Arntl* and *Nr1d1* lost their rhythms, whereas *Cry2* rhythms were robust after IH exposure. No detectable rhythms of *Clock*, *Per3*, and *Npas2* were identified in both conditions. This may be a cause of multiple disparate cell types in the whole brain and/or the sampling resolution in this tissue. We also observed an elevated midline expression level of *Per1* and *Per2* in normoxia vs. IH (Supplementary Table S1).

In the liver, there are a higher number of rhythmic genes compared to any other peripheral tissue [14]. The disrupted clock in the liver strongly influences the transcriptional rhythms of other peripheral tissues [15]. Therefore, we measured the circadian rhythms of the canonical clock genes in liver samples (Figure 2B). Eight out of ten canonical genes (*Arntl*, *Cry1*, *Per1*, *Per2*, *Per3*, *Npas2*, *Dbp*, and *Nr1d1*) were rhythmic under both conditions. However, *Per1* and *Nr1d1* were reduced in the amplitude and midline expression in response to IH (Supplementary Table S1). Furthermore, the *Clock* lost its rhythmicity after IH exposure. In addition, the *Cry2* rhythm was not detected in both normoxia and IH conditions. This was in line with other large-scale rhythmic expression datasets [16]. Overall, our observations suggested that IH alters the tissue-specific rhythms of canonical clock genes.

### 3.2. The Clock in the Liver Is More Stable against IH Compared to the Clock in the Brain

**Figure 3:**
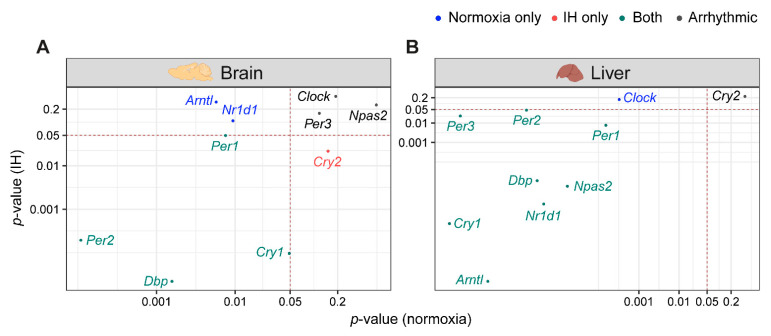
IH alters rhythmic expression of clock genes. Correlation plots represent rhythmicity *p*-values between normoxia vs. IH in brain (**A**) and liver (**B**). Genes rhythmic only in normoxia, only in IH, and in both are represented in blue, red, and cyan, respectively. The number of rhythmic clock genes in both conditions suggests that the circadian clock in the liver is less sensitive to IH compared to the circadian clock in the brain. Arrhythmic genes in both tissues are represented in black.

Light–dark cycles and the availability of food are two important zeitgebers (environmental signals) that regulate the mammalian circadian system. The clocks in the brain are directly responsive to light whereas the clocks in the liver are responsive to diet. The functional co-ordination between the brain and the liver plays a significant role in the maintenance of the circadian system for the adaptation and survival of mammals [17]. Therefore, we compared the influence of IH on the clock genes in the liver and the brain using *p*-value correlation plots (Figure 3). Seven out of ten genes in the brain (Figure 3A) and nine out of ten genes in the liver (Figure 3B) were rhythmic in at least one of the normoxic or IH conditions. Among these rhythmic genes, eight genes in the liver and four genes in the brain did not respond to IH. These results suggested that the circadian rhythms of the clock genes in the liver (Figure 3B) were more stable after exposure to IH compared to brain (Figure 3A).

## 4. Discussion

At least one-third of adults in the United States experience IH as seen in sleep-related breathing disorders [18]. These conditions can impact the circadian clock [18,19,20]. We sought to understand the impact of IH on the canonical clock genes of two important tissues (the brain and the liver) involved in the regulation of the mammalian circadian system.

So far, only two other studies have evaluated the impact of IH on time- or tissue-dependent gene expression under diurnal conditions [9,10]. These studies were limited to the peripheral tissues with the analysis of only a few clock genes. We evaluated the circadian rhythms in 10 canonical clock genes from central and peripheral tissues in mice after normoxia or IH exposure. This isolated the impact of IH on the circadian system.

Previous studies showed that the circadian clock in the mouse liver was less sensitive to hypoxia exposure compared to the other peripheral organs [9]. Interestingly, we observed fewer circadian changes in the liver compared to the brain, a central organ not previously studied in this context. Future studies are needed to understand the clinical consequences of the tissue-specific responses to IH which accompany a host of diseases.

## 5. Limitations

Our study is limited to the rhythms of the clock genes in the whole brain rather than the specific regions of the brain. Factors such as the sampling resolution may influence the rhythmic expression patterns in heterogenous tissues. However, these limitations do not undermine our observations as the brain as a whole organ is sensitive against hypoxia [13]. The evaluation of the whole-organ responses to hypoxia are more applicable to our current study. The current study is mainly focused on the canonical clock genes in the brain and the liver. However, a comprehensive transcriptome study should be performed to explore the tissue-specific differential rhythms in multiple organs. Our study does not describe the functional consequences of altered gene expression. As with any animal model, our IH model does not exactly replicate diseases or the associated sequelae. We used three mice per time point. We cannot discount the inter-individual variability in the response to IH; therefore, increasing the number of animals per time point may be helpful in further studies.

## 6. Conclusions

In summary, our results reinforce the concept that the clock response to IH is tissue-dependent. The organ sensitivity to IH can play a significant role in the alteration of circadian rhythms in the brain. Our observations also provided insights to potential underlying etiologies of circadian rhythm-associated health conditions.

## Figures and Tables

**Table 1:** TaqMan^®^ Gene Expression assay IDs.

Gene Name	Assay ID
*Arntl*	Mm00500226_m1
*Clock*	Mm00455950_m1
*Cry1*	Mm00514392_m1
*Cry2*	Mm01331539_m1
*Dbp*	Mm00497539_m1
*Npas2*	Mm00500848_m1
*Nr1d1*	Mm00520708_m1
*Per1*	Mm00501813_m1
*Per2*	Mm00478113_m1
*Per3*	Mm00478120_m1
*Gapdh*	Mm99999915_g1
*Ppib*	Mm00478295_m1

## Data Availability

The data presented in this study are available on request from the corresponding author.

## Supplementary Materials

The following are available online at https://www.mdpi.com/article/10.3390/genes12101627/s1, Table S1: Differential rhythms of canonical clock genes between normoxia and intermittent hypoxia.
